# Validation of a Cost-Efficient Multi-Purpose SNP Panel for Disease Based Research

**DOI:** 10.1371/journal.pone.0019699

**Published:** 2011-05-17

**Authors:** Liping Hou, Christopher Phillips, Marco Azaro, Linda M. Brzustowicz, Christopher W. Bartlett

**Affiliations:** 1 Battelle Center for Mathematical Medicine, The Research Institute at Nationwide Children's Hospital, Columbus, Ohio, United States of America; 2 Forensic Genetics Department, Genomic Medicine Group, University of Santiago de Compostela and Centro Nacional de Genotipado (CeGen), Genomic Medicine Group, Hospital Clínico Universitario, Galicia, Spain; 3 Department of Genetics, Rutgers University, New Brunswick, New Jersey, United States of America; 4 Department of Pediatrics, The Ohio State University, Columbus, Ohio, United States of America; South Texas Veterans Health Care System, United States of America

## Abstract

**Background:**

Here we present convergent methodologies using theoretical calculations, empirical assessment on in-house and publicly available datasets as well as *in silico* simulations, that validate a panel of SNPs for a variety of necessary tasks in human genetics disease research before resources are committed to larger-scale genotyping studies on those samples. While large-scale well-funded human genetic studies routinely have up to a million SNP genotypes, samples in a human genetics laboratory that are not yet part of such studies may be productively utilized in pilot projects or as part of targeted follow-up work though such smaller scale applications require at least some genome-wide genotype data for quality control purposes such as DNA “barcoding” to detect swaps or contamination issues, determining familial relationships between samples and correcting biases due to population effects such as population stratification in pilot studies.

**Principal Findings:**

Empirical performance in classification of relative types for any two given DNA samples (e.g., full siblings, parental, etc) indicated that for outbred populations the panel performs sufficiently to classify relationship in extended families and therefore also for smaller structures such as trios and for twin zygosity testing. Additionally, familial relationships do not significantly diminish the (mean match) probability of sharing SNP genotypes in pedigrees, further indicating the uniqueness of the “barcode.” Simulation using these SNPs for an African American case-control disease association study demonstrated that population stratification, even in complex admixed samples, can be adequately corrected under a range of disease models using the SNP panel.

**Conclusion:**

The panel has been validated for use in a variety of human disease genetics research tasks including sample barcoding, relationship verification, population substructure detection and statistical correction. Given the ease of genotyping our specific assay contained herein, this panel represents a useful and economical panel for human geneticists.

## Introduction

While microarray SNP genotype data are the standard genotype data in human genetics there will still be a financially motivated need for lower throughput genotyping to accomplish a range of tasks prior to larger investments. Thus when dense whole genome SNP or whole genome sequence data are not available, as is the case in pilot studies and other instances when further genotyping would be cost prohibitive (e.g. targeted measured genotype approaches on very large population samples or follow-up genotyping on a replication cohort after a large discovery phase), it is more economic to use a small number of SNPs for: 1) Unique barcoding of DNA samples for quality assurance to detect sample swaps and contamination; an important step even in small pilot studies. 2) Verification of family relationships even in small structures such as parent-child trios is important given a general non-paternity rate estimated to be ∼3% [1]. 3) Adjustment of case-control association sample sets to control for potential type I error when case and control samples may systematically differ in their genetic backgrounds based on population substructure [2]. In this case, it is possible to perform either analysis that uses quantitative admixture information as a covariate to return the statistical test of size to nominal levels, or analyze subsets of the data based on genetic background and use a meta-analysis or other method to combine information across the populations. Either correction method requires genome wide information for the quantification or detection, respectively, of population substructure.

Butler et al. [3] summarized a discussion of the 2007 meeting of International Society of Forensic Genetics including a classification of SNP markers based on the type of information provided by the SNP. Forensic investigations often seek to uniquely identify a DNA sample and to assign a DNA sample to an ancestral group. The two goals require different classes of SNPs. “Individual identification SNPs” have high heterozygosity while “ancestry informative SNPs” have high differentiation between populations commonly measured by F_ST_. These two goals, taken to their logical extremes, are necessarily mutually exclusive since the highest F_ST_ values come from variants that are fixed in different populations for opposite alleles (i.e., little or no heterozygosity in any single population). Thus, if a small number of SNPs is being chosen for a given purpose, those SNPs may not have desirable properties for both individual identification and ancestry. For example, Kidd et al [4] selected a panel of 19 SNPs that were specifically chosen to minimize F_ST_ to create a universal SNP panel for individual identification that was not dependant upon ancestry. The 19 SNP panel achieves its stated goal and is therefore not informative for ancestry. On the other end of the spectrum, a 93 SNP panel was selected for large F_ST_ values by Nassir [5] that is useful for ancestry assignment, but may not be as useful for personal identification except that some of the 93 SNPs provide some information on individual identity since a proportion show some heterozygosity in certain populations.

Recently, a panel of 52 SNPs was selected for use in forensic investigations [6] with the stated goal of individual identification across populations [6], [7], [8], [9], [10], [11], [12]. This particular panel is promising as the number of SNPs is easily multiplexed in one or at most two assays for processing which makes this scale of marker set ideal in terms of both cost and information content. Tracking samples in a human disease genetics lab by obtaining unique profiles of polymorphic markers (sp called ‘barcoding’) is akin to comparing sample identity to a reference sample, except for human disease genetics applications the reference sample is the genotypes obtained when the sample was first processed in the lab and deviations from that reference profile highlight quality assurance issues. In addition, the selection of the 52 SNP panel, while centering on SNPs with high overall heterozygosity for most populations, also incuded a smaller proportion of SNPs showing highly contrasting allele frequency distributions in particular populations; raising the possibility that this SNP panel would also be useful to detect and correct for population admixture. Lastly any panel that is informative for individual identification in different human populations is also applicable to relationship testing provided that the majority of the composite SNPs are unlinked - as is the case of the 52 SNP panel.

We sought to test these properties empirically, by examining the 52 SNP panel applied to a variety of common human genetics tasks and analysis methods to verify the utility of this panel in these instances. We used a combination of publicly available data as well as genotypes generated in-house through a set of two multiplexed assays for these SNPs to assess the suitability of the panel for barcoding, relationship checking and admixture detection. Additionally, since it is unclear if enough Ancestry Informative Markers (AIMs) were included in the 52 panel to statistically control false positive rates for case-control association analysis either quantitatively or through stratifying the sample based on identified subgroups, we conducted a simulation study to determine the approximate range of population stratification between European and African populations where this panel would be most useful. We compared the performance of the 52 SNP panel with two other SNP panels from the literature comprising 19 and 93 SNPs [4], [5]. The 19 SNP panel represents the minimum amount of information for optimal individual identification. In contrast, the 93 SNP panel consisted of AIMs selected to identify the continental ancestries of subject groups for genetic studies and demonstrated to be efficient for that purpose. We hypothesized that the 52 SNP panel would perform in a comparable way to the 19 SNP panel for identification but would be unlikely to match the performance of the 93 SNP AIM panel for assignment of ancestry since this was not the primary purpose of the 52 SNP panel in the first place. We also gauged the performance of the 52 SNP panel for routine relationship verification (relationship checking) in outbred extended pedigrees.

## Results

The mean match probabilities obtained with the 52 SNP panel for the CEPH pedigrees (1.97×10^−19^) and HapMap samples (CEU 4.14×10^−20^; YRI 2.59×10^−15^; CHB 4.25×10^−17^; JPT 1.27×10^−16^) were in line with previous estimates [4], [6]. Even though the CEPH pedigree sample contains related individuals, the mean match probability is still lower than any non-European population by 2 orders of magnitude. This reflected a slight ascertainment bias in the SNP selection process where about 15% of loci originally had population data for Europeans alone and in several cases these showed later to have much less variability in African and/or East Asian populations. When examining the Human Genome Diversity Panel (HGDP) data from seven populations the mean match probabilities were between 10^−13^ and 10^−18^. With Europe and Southwest Asia being the lowest and sub-Saharan Africa the highest, a trend mirrored in the HapMap samples. We note that certain markers in the 52 SNP panel are sited on the same chromosome arm and have recombination rates below 0.4, but no markers showed evidence of linkage disequilibrium in any sample. Dropping the least informative markers of any same-arm set reduces the panel to 42 SNPs and increased the mean match probability by no more than 3.3 orders of magnitude and no less than 1.7 for each population. Therefore any one profile for a given population (whether based on a subset reduced to allow for linkage or the full set) will have a match probability lower than the world population of ∼7×10^9^, representing a globally unique profile amongst unrelated individuals.

Table 1 presents the summary statistics for the distribution in the number of matches for all possible pairs in each sample. The distribution of matches was normally distributed in each sample as indicated by central moments of the distributions including skewness and kurtosis. The number of comparisons that comprised the distributions are also listed as a general indicator of the relative information contained in each distribution. The greatest number of matches occurred in the CEPH pedigree sample, which is the only sample to contain related individuals, though the average number of matches is similar across all groups.

**Table 1:** Descriptive statistics on the number of identical genotypes between all possible pairing of samples by group.

	CEPH Pedigrees	CEU HapMap	YRI HapMap	CHB HapMap	JPT HapMap	HGDP
Mean	21.9	19.5	24.7	22.2	22.7	17.1
SD	5.3	3.6	3.4	3.4	3.4	3.6
Skewness	0.19	0.14	0.14	0.12	0.15	0.07
Kurtosis	0.59	0.04	0.15	−0.03	−0.25	0.07
Minimum	4	8	13	12	13	2
Maximum	43	34	41	33	32	35
Number of comparisons	3828	3240	3240	903	861	452676

The CEPH pedigrees showed no evidence of misspecified relationships or sample swaps based on RELCHECK analysis. We calculated the difference between the most likely (and in this case, correct) relationship and the next most likely relationship as a measure of relationship resolving power. The average difference was a likelihood ratio of 4.2 (SD = 14.0, range 1.1 to 302.0). Upon visual inspection most of the lower likelihood ratios were the parent-child to half-sibling contrasts. Thus while low likelihood ratios are not encouraging for the SNP panel to resolve relationships, this particular contrast would cause Mendelian inconsistencies if inappropriate adjustments were made to the pedigree file, thus ensuring such a mistake would not be made. Other relationship contrasts were much more readily resolved with average likelihood differences of 2.5×10^14^ and 1.2×10^54^ for the third and fourth most likely relationships versus the most likely relationship.

**Figure 1:**
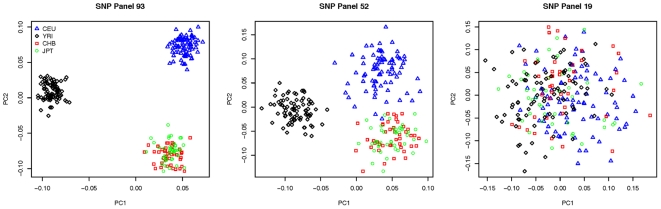
Detection of population structure in four HapMap populations. The first two principal components from EIGENSTRAT are plotted for all 3 SNP panels (A, SNP panel 93; B, SNP panel 52; C, SNP panel 19). As more AIMs are used in the analysis, the resolution improves. The 52 SNP panel appears to have some overlap between CEU and CHB+JPT though it should be noted that these datapoints are more clearly differentiated by considering the third and fourth principal components (not shown).

Figure 1 shows the sensitivity of each marker set for detecting population admixture. The figure supports previous data showing that the 52 and 93 SNP panels can be used to efficiently identify continental groups of Europe (CEU), Asia (CHB and JPT) and Africa (YRI), though neither can differentiate CHB and JPT population samples [5], [12]. There was minimal overlap between CEU and Asian (CHB+JPT) for the 52 SNP panel, and no overlap between CEU and YRI populations. As expected, the 19 SNP panel fails to distinguish any populations based on these data.

**Figure 2:**
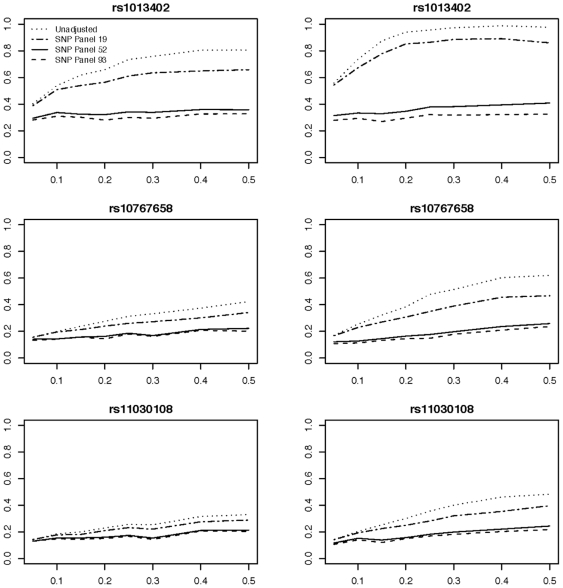
Analysis of case-control study type I error rates from 3 simulated SNPs within *BDNF*. The three SNPs show allele frequency differences between CEU and YRI of 0.066 (rs11030108), 0.102 (rs10767658), and 0.233 (rs1013402). The y-axis is estimated type I error rate versus the simulated CEU proportion (x-axis). Panels on the left show data with a difference in disease prevalence ratio of 1.25 while a ratio of 1.5 is shown on the right.

**Figure 3:**
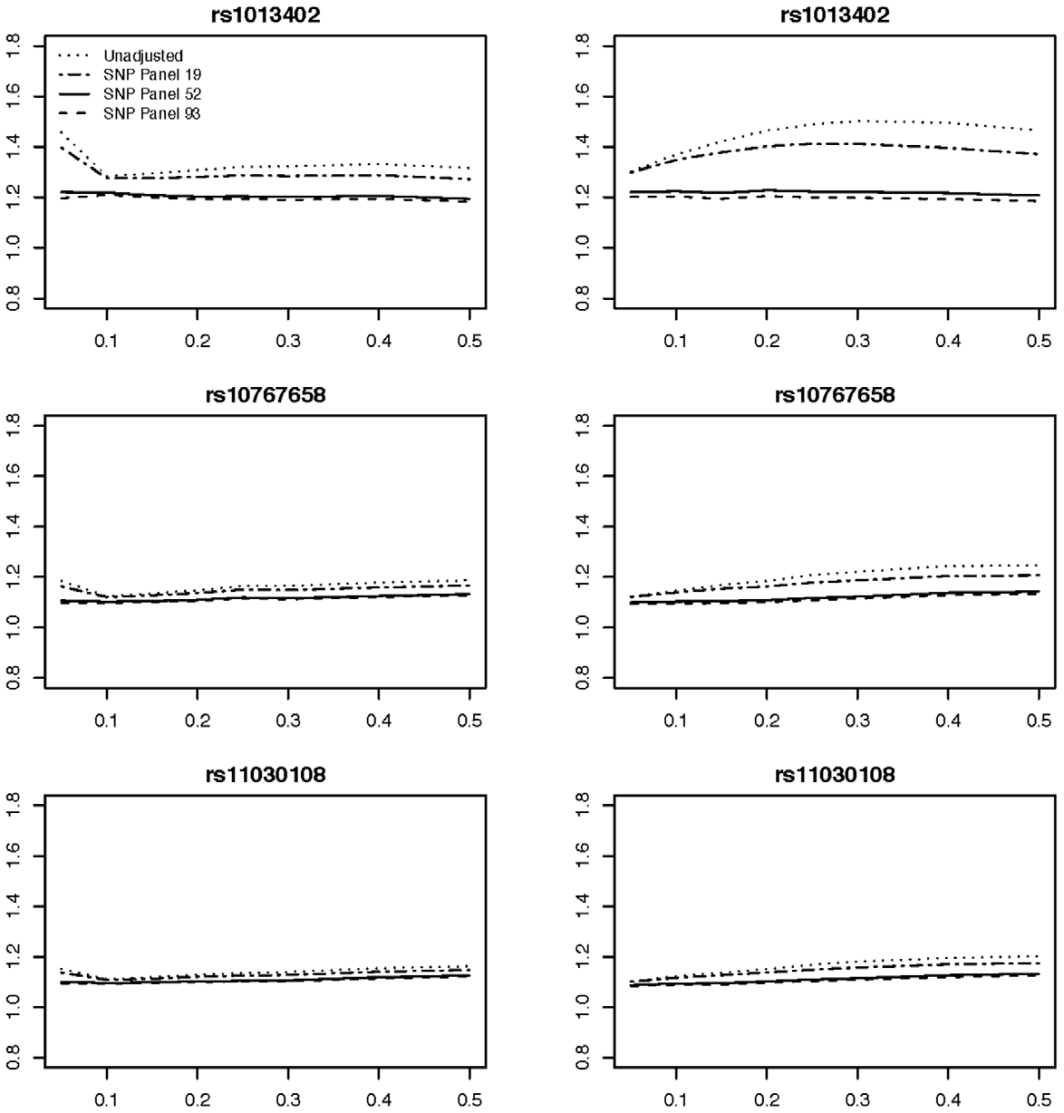
Estimated odds ratios (OR) from case-control analysis of 3 simulated SNPs within *BDNF*. Conventions are the same as Figure 2 except the y-axis is the average estimated OR from the same analysis as presented in Figure 2.

We also simulated population stratification in a case-control study design by using a mixture of CEU and YRI haplotype frequencies, a difference in population disease rates, and by varying the proportion of CEU haplotypes in each replicate. Figures 2 and 3 shows the results when using no adjustment for population stratification compared to analysis with adjustment by listing type I error and estimated OR (real OR is always 1 under null simulations). Under the 1.25 CEU-YRI disease prevalence difference condition, both the 52 and 93 SNP panels performed well in terms of type I error and estimated OR. When looking at the more extreme 1.5 CEU-YRI disease prevalence difference condition both panels continue to perform well, though the 93 SNP panel showed a slight advantage in absolute terms, but the difference in estimated OR (or type I error rate) is still trivial thus demonstrating the sufficiency of information from the 52 SNP panel to statistically correct for population stratification over a wide range of parameters.

## Discussion

We examined a panel of 52 SNPs, originally selected for utility in forensic investigations, to determine if this panel would also be useful for human genetics researchers in a variety of common applications in that field. Consistent with the original work on this panel [6], we found mean match probabilities comparable to those previously observed in four populations from the three continental groups most commonly used as subjects in genetic analyses. In six large pedigrees we found very similar distributions of identical genotypes between pairs and no sample pair showed identical profiles. Since pedigrees contain related individuals this confirms the panel is sufficiently heterozygous to differentiate close relatives though more evaluations would be needed for those pedigrees with loops and significant levels of inbreeding. Consistent with the low mean match probability and our observations in pedigrees, the panel is clearly able to deliver a globally unique profile or barcode for tracking samples and enable straightforward quality assurance in human genetics research.

Our study also goes beyond the original characterization of the 52 SNP by Sanchez et al. [6] in several key ways. Firstly, we assessed the ability of the panel to resolve complex relationships by examining CEPH pedigrees with large sibships and three generations. This covers the most common pedigree relationships encountered in the human genetic literature. Exact likelihood calculations provided a mechanism to quantify the resolving power of the panel, which is far superior to the 19 SNP panel and only marginally less efficient than choosing 500 SNPs with high minor allele frequency uniformly distributed across the genome, a procedure used in the many extended pedigrees projects in our lab (data not shown).

Secondly, while Sanchez et al. [6] used classical hierarchical clustering methods common in phylogenic analysis (Unweighted Pair Group Method with Arithmetic Mean) to assess the ability of the panel to differentiate ancestries, in the human disease discovery field this methodology is uncommon. Here we have applied PCA to identify population structure using EIGENSTRAT, a common statistical package used in human disease discovery projects. Our results are qualitatively similar to the hierarchical clustering of Sanchez et al. [6] which, based on different DNA samples, also showed the 52-SNP panel was able to distinguish 3 major populations, that of European, African and Asian. Thirdly, and perhaps most importantly, we examined if the panel provided enough information about population structure to quantitatively correct the effect of admixture in case-control association analysis. The data support the conclusion that population stratification between CEU and YRI populations can effectively be corrected with this panel though when such corrections are inadequate, it is at least possible to detect that admixture is present.

This study is limited by the data structures examined and our modeling assumptions for the simulations. In the former case, we have assessed the information content of this panel to identify more distant relationships than avuncular. It would be expected that half-sib relationships and cousins would be reasonable to detect with this panel but more distant relationships would clearly be more difficult to resolve while inbreeding and marriage loops may not be sufficiently differentiated from sibling or parent-child relationships to be reliably detected. Additionally, since the data presented here is based on slightly fewer SNPs than the full panel (48 for HapMap data or 49 for our CEPH family genotyping) the resolving power of the panel is marginally underestimated. It is worth noting that the inclusion of dedicated ancestry informative marker SNPs such as rs1426654 (fixed in Europeans) or rs2814778 (fixed in Africans) to augment the above identification loci would further strengthen the ability of this multiplex to differentiate the major population groups [12].

For evaluating false positives in case-control analysis, we only simulated a limited number of disease models and while the simulations are realistic for the relationship between the SNPs for each population (due to use of real genotype sets), it is clear that simple combinations of populations differing in cases and controls is not a fully realistic simulation of stratification in real populations. However, it is clear that our model does capture the end result of continuous and gradual admixture, which is most important for understanding if the SNP panel presents a viable strategy for statistical correction.

We have evaluated a panel of 48 SNPs (derived from a published set of 52) and presented a method for two multiplex PCR/LDR assays for those SNPs. This panel of SNPs is suitable for sample quality assurance in many human genetics research lab in the form of sample barcoding to detect sample swaps and contamination as demonstrated by the extremely low probability of two samples having identical profiles. Relationship checking in simple extended pedigree structures is clearly possible given the resolving power of the SNP panel, although the effect of the reduced variability when inbreeding or marriage loops may be present in the pedigree or when analyzing more distant relationships beyond cousin pairs, remains to be explored. Consistent with previous work on population inference with this panel, three broad population groups may be readily distinguished (European, African, East Asian) and within those broad categories several subgroups are also distinguishable. However, the purpose of the panel as presented here for human disease genetics is not to assess samples for categorizing into population groups, rather the purpose is to capture variance attributable to different populations that is necessary to correct for population stratification in case control studies. This panel indicates it can provide that level of information over a range of disease models and population stratification scenarios.

## Methods

### Samples

For testing population identification we used publicly available samples and genotyping data selected from HapMap [13] (Phase 3) for European sample: CEU (Utah residents with ancestry from northern and western Europe), African: YRI (Yoruba in Ibadan, Nigeria), Japanese: JPT (Japanese in Tokyo, Japan) and Chinese: CHB (Han Chinese in Beijing, China). Genotype data for the composite markers of the three different SNP panels was downloaded from the HapMap website (http://hapmap.ncbi.nlm.nih.gov/). The first SNP panel, developed by Kidd and colleagues [4], comprises 19 SNPs, with 17 characterised in all four populations of HapMap (rs1358856 and rs7520386 lack data). From the 1000 Genome Project data, we found a proxy SNP rs1358855 for rs1358856 (pairwise r^2^ between these two SNPs was 0.97, 1.0 and 1.0 for CEU, CHB+JPT and YRI respectively). The second SNP panel of 52 SNPs, previously developed for forensic analysis by the SNP*for*ID Consortium [6], of which 48 can be found in all of 4 populations (rs826472, rs2016276, rs938283 and rs722098 lack data). All 93 SNPs of the third AIM-SNP panel [5], are characterized in all 4 HapMap populations, though only a subset of these SNPs was genotyped for HapMap phase 3 individuals, so only phase 2 population data was used for ancestry analyses we performed. After quality control (removal of individuals with greater than 5% missing genotypes), the sample sizes for CEU, YRI, CHB and JPT reduced to 81, 81, 43 and 42 respectively. For mean match probability, we also used data from the Human Genome Diversity Panel [14], divided into seven continental groups Oceana, South Asia, East Asia, Sub-Saharan Africa, North and South America, and Europe. A total of eight SNPs (rs1029047, rs2107612, rs873196, rs1382387, rs2111980, rs938283, rs1028528, rs1528460) cannot be found in all continental groups from HGDP files and were not used for calculation.

For gauging the ability of SNP sets to resolve relationships we genotyped a series of families from the Centre d"Etude du Polymorphisme Humain (CEPH) collection [15] available through The Coriell Cell Repositories http://ccr.coriell.org. Each family consists of a large sibship plus both parents and both sets of grandparents available for genotyping. Family numbers were 1451, 1454, 1456, 1458, 1459 and 1463 with a total of 88 samples for genotyping.

### Genotyping

Single nucleotide polymorphism genotyping: Genotyping was performed by multiplex PCR of amplicons containing the 52 SNPs followed by the ligase detection reaction (LDR) method of Bruse et al. [16] on a Luminex 200 Multiplex Bio-Assay Analyzer. Assay details are presented in Table 1. All samples were stored and handled as described previously [17]. Genotypes were called based on the same metric as Bruse et al [16] but initial clustering analysis was conducted by in-house Ruby 1.8.6 scripts applying the AI4R library (http://ai4r.rubyforge.org/) clustering routines and the Tioga graphics library (http://www.itp.ucsb.edu/~paxton/tioga.html). This script automatically produces allele intensity plots color coded by called genotype. All plots were visually inspected (by CWB); SNPs that did not cluster appropriately were dropped (N = 3; rs733164, rs907100, rs938283), as this is indicative of an assay failure, leaving a total of 49 of 52 SNPs with robust genotype information.

Genotype cleaning: All genotype data was automatically processed once generated via in-house Ruby 1.8.6 scripts for consistency and to minimize human errors. Files containing the called genotypes were output to a pre-MAKEPED linkage format file [18]. A linkage format locus data file was generated to form the two required inputs to PEDCHECK v1.1 [19] which detects all Mendelian inconsistencies. PEDCHECK output was tabulated and merged with raw allele intensity data for manual evaluation of the genotype calls in the flagged nuclear family containing the error. Ambiguous genotypes were repeated (N = 9 out of a total of 4312 genotypes). No genotypes were excluded after repeating. PEDSTATS v0.6.3 was used to check markers for Hardy-Weinberg equilibrium using the founders only functions [20]. No markers were flagged as deviating from Hardy-Weinberg equilibrium at the P<0.05 level. No SNPs showed significant linkage disequilibrium (D') in either the HapMap data or in our CEPH pedigrees as determined by maximum likelihood estimation.

### Simulations

HAP-SAMPLE [21], a data based resampling simulation tool, was used to simulate genetically realistic genotypes for SNPs in the three panels plus SNPs in a candidate gene under a null model (in this case, brain-derived neurotrophic factor or *BDNF*). HAP-SAMPLE is based on a pool of phased chromosomes estimated from genotyped HapMap samples. Using a resampling approach that assumes random mating and implements crossovers as part of simulated meiosis, HAP-SAMPLE can be used to create artificial case-control samples of arbitrary size that possess realistic patterns of linkage disequilibrium and genotype distributions. In our study, simulations proceeded as follows: firstly, 10,000 CEU cases and 10,000 CEU controls were generated based on HapMap CEU data, and 10,000 YRI cases and 10,000 YRI controls were generated based on YRI data, secondly, 500 cases and 500 controls were randomly selected from CEU & YRI cases and CEU & YRI controls, respectively. The specific number of samples selected from each group (CEU cases, CEU controls, YRI cases, YRI controls) was determined by two factors, 1) CEU proportion and 2) degree of population stratification defined as the ratio of disease prevalence between CEU and YRI populations being greater than 1. For example, if the CEU proportion in the final sample is 50% and under moderate population stratification (the prevalence of the disease was 1.25 fold higher in CEU than YRI), then the CEU cases should be 278, CEU controls should be 222, YRI cases should be 222, YRI controls should be 278. Each combination of the following parameter sets was simulated 1000 times. CEU proportion was varied in the set [0.05, 0.1, 0.15, 0.2, 0.25, 0.3, 0.4, 0.5]. Ratio of disease prevalence CEU∶YRI was either 1.25 or 1.5.

### Statistics

Mean match probability was calculated as the sum of the squared genotype frequencies per locus. This procedure was applied twice, once using all of the markers and once excluding the less informative of any two SNPs that were estimated to have a recombination fraction <0.4 (i.e, linked) for total of N markers dropped. Using estimated genotype frequencies for HapMap samples mean match probability was calculated by population and identically estimated for the in-house genotyped CEPH pedigrees. Relationship testing performance was checked using RELCHECK, which estimates identity by state (IBS) relationship values between each pair of individuals. A log_10_ likelihood (LOD) score is calculated over 5 contrasting relationships (parent-child, full sibling, half sibling, monozygotic twin and unrelated). The denominator of the LOD score for all evaluations within a sample pair is the most likely pairwise relationship. LOD differences between the most likely relationship and the correct relationship were calculated. If this value was zero, the difference between the correct relationship and second most likely relationship was used a measure of resolving power.

Populations were resolved by principal component analysis of the SNP genotype data as implemented in EIGENSTRAT [22]. EIGENSTRAT was used to calculate the principal component analysis (PCA) scores for each SNP panel, and the first 4 principal components were put into a logistic regression model to adjust for population stratification in the simulated data. We compare analysis of the data with and without the population stratification adjustment by plotting changes in type I error and estimated odds ratio (OR).
